# Efficacy of Fosfomycin-Containing Regimens for Treatment of Bacteremia Due to Pan-Drug Resistant *Acinetobacter baumannii* in Critically Ill Patients: A Case Series Study

**DOI:** 10.3390/pathogens12020286

**Published:** 2023-02-09

**Authors:** Stelios F. Assimakopoulos, Vassilis Karamouzos, Gerasimos Eleftheriotis, Maria Lagadinou, Christina Bartzavali, Fevronia Kolonitsiou, Fotini Paliogianni, Fotini Fligou, Markos Marangos

**Affiliations:** 1 Division of Infectious Diseases, Department of Internal Medicine, University of Patras Medical School, 26504 Patras, Greece; 2 Department of Anaesthesiology and Intensive Care Medicine, University of Patras Medical School, 26504 Patras, Greece; 3 Department of Microbiology, University of Patras Medical School, 26504 Patras, Greece

**Keywords:** *Acinetobacter baumannii*, pan-drug resistant, bacteremia, ICU, colistin, fosfomycin

## Abstract

*Acinetobacter baumannii* (AB) has evolved over the last decades as a major problem in carbapenem-resistant gram-negative nosocomial infections, associated with high mortality rates especially in the intensive care unit (ICU). Recent reports highlight the increasing prevalence of resistance to colistin, a last resort therapeutic option for carbapenem-resistant AB. We retrospectively evaluated the characteristics, treatment regimens and outcomes of twenty patients with pan-drug resistant (PDR) AB primary bacteremia hospitalized in the ICU of the University General Hospital of Patras, during a two-year period (October 2020–September 2022). The 28-day mortality reached 50%. Between survivors and non-survivors, no differences were found regarding age, gender, and Charlson comorbidity index (CCI). However, non-survivors had higher APACHE II scores and higher prevalence of septic shock and COVID-19 infection. A significantly higher percentage in the survivor group received Fosfomycin as part of the combination regimen. Inclusion of fosfomycin in the combination therapeutic regimen was associated with significantly better survival as compared to non-fosfomycin-containing regimens. In view of the increasing prevalence of PDR-AB infections in ICUs, its associated high rates of mortality and the lack of effective treatment options, the observed survival benefit with fosfomycin inclusion in the therapeutic regimen merits further validation in larger prospective studies.

## 1. Introduction

*Acinetobacter baumannii* (AB) has evolved over the last decades as a major problem in multidrug resistant gram-negative nosocomial infections, especially in the intensive care unit (ICU). Its intrinsic antimicrobial resistance, together with its ability to easily adopt new resistance mechanisms, has driven the evolution of extensively drug-resistant (XDR) and even pandrug-resistant (PDR) isolates [1]. In Greece, data reported in the Electronic System for the Surveillance of Antimicrobial Resistance (WHONET-Greece) for the three-year period from January 2018 to March 2021, demonstrate that, steadily, more than 95% of the isolates are carbapenem-resistant [2]. Colistin represents the cornerstone of therapy, but colistin resistance is an evolving problem, while PDR strains are also increasing worldwide, especially in ICUs. AB blood isolates from hospitalized patients in Greek ICUs present high rates of colistin resistance, ranging from 27.5–57.8% in the pre-pandemic period (increasing trend) to 53–47% during the pandemic (decreasing trend) [2].

We present, herein, our tertiary centre experience with 20 ICU patients suffering from bacteremia due to PDR-AB. PDR-AB strains were defined as carbapenem- and colistin-resistant with high tigecycline MICs > 2 μg/mL. Although this is a small case series of 20 patients, the importance of the problem of PDR-AB infections in Greek ICUs, the associated significant excess mortality, and the lack of treatment options except from synergistic combinations with limited clinical evidence point towards the potential value of this study [3,4,5]. The reported patients received diverse salvage combination regimens, which were recorded in conjunction with epidemiological data, clinical severity scores, microbiological and clinical outcomes.

## 2. Patients and Methods

We retrospectively evaluated the characteristics, treatment regimens and outcomes of patients with PDR-AB primary bacteremia hospitalized in the ICU of the University General Hospital of Patras, Greece, a 770-bed teaching hospital, during a two-year period (October 2020–September 2022). The study was approved by the Ethical Committee of the University General Hospital of Patras; need for informed consent was waived (No 858). Patients were identified using the database of the microbiology department. AB strains isolated from blood cultures deriving from ICU patients were identified using the Vitek 2 Advanced Expert System (bioMerieux, Marcy l’Etoile, France). Antibiotic susceptibility was performed by the Vitek 2 Advanced Expert System, while MIC to tigecycline was determined by Etest (AB Biodisk) and MIC to colistin by broth microdilution method. Cefiderocol and eravacycline were not tested because these antibiotics are not readily available in Greece. Patients with culture-proven polymicrobial infections were excluded. Results were interpreted according to EUCAST guidelines (EUCAST, 2023). PDR phenotype was defined according to the international expert proposal for Interim standards guidelines [6]. Accordingly, PDR-AB isolates were non-susceptible to all agents in all antimicrobial categories, with tigecycline MICs > 2 μg/mL. Regarding tigecycline, in the latest EUCAST clinical breakpoints (v. 13.0, 1 January 2023) no clinical breakpoint is set due to insufficient evidence [7]. However, previous clinical studies with XDR-AB infections, have shown that when tigecycline MIC is >2 μg/mL, significantly higher therapeutic failures and mortality were observed either when tigecycline was used as monotherapy or as part of a combination therapy with colistin [8,9]. In addition, the clinical efficacy of tigecycline is reached when *ƒ*AUC_0–24h_/MIC ratios are greater than 0.9, which cannot be achieved, especially in bloodstream infections, even with high doses of tigecycline (200 mg loading dose followed by maintenance dose 100 mg b.i.d) [10]. The therapeutic regimen used in each patient was defined after infectious diseases consultation. The usual antimicrobial dosages adopted for the most used antibiotics were as follows: for colistin, a loading dose of 9 million IU followed by 4.5 million IU every 12 h; for tigecycline, a loading dose of 200 mg followed by 100 mg every 12 h; for gentamicin, a dosage of 5–7 mg/kg every 24 h; for amikacin 15–20 mg/kg every 24 h; for meropenem, a dosage of 2 g every 8 h in 3 h infusion; for piperacillin/tazobactam a dosage of 4.5 g every 6 h in 3 h infusion; for ampicillin/sulbactam 9 g every 8 h in 4 h infusion; for trimethoprim/sulfamethoxazole 5 mg TMP component/kg every 12 h; for fosfomycin 8 g every 8 h in 3 h infusion. Epidemiological data, extent of comorbid illnesses defined by the Charlson comorbidity index (CCI), severity of illness scores Acute Physiology and Chronic Health Evaluation II (APACHE II) and Sequential Organ Failure Assessment (SOFA), antibiotic regiments, microbiological response, and 28-day all-cause mortality were obtained from patients’ chart reviews and the ICU computerized database (Criticus™, University of Patras, Patras, Greece). APACHE II and SOFA score were determined on the day that the positive blood culture was drawn. Septic shock was defined according to international definitions [11]. Microbiological success was defined as a follow up negative culture for AB at days 7 or 14.

Data analyses were performed by SPSS version 28.0 (SPSS Inc., Chicago, IL, USA) software. All variables were tested for normal distribution using the Kolmogorov–Smirnov test. The comparison of continuous variables was calculated by two sample *t*-test (normally distributed variables) or Mann–Whitney *U*-test (non-normally distributed variables). The comparison of the categorical variables was calculated by chi-square test with Yates’ correction if required. Survival outcomes were assessed using the Kaplan–Meier curve analysis. All statistical tests were two-tailed and *p* < 0.05 was considered statistically significant.

## 3. Results

The present study included 20 ICU patients with bacteremia due to PDR-AB. Patients had a mean age of 62.4 ± 14.2 years, 55% of them had a CCI ≥ 3. The characteristics of the studied patients and their clinical and microbiological outcomes are presented in Table 1.

In our cohort, 28-day all-cause mortality reached 50%. Between survivors and non-survivors, no differences were found regarding age, gender, and CCI. Non-survivors had higher APACHE II (25.6 ± 10.8 vs. 16.3 ± 6.6, *p* = 0.032) and SOFA (11.4 ± 4.3 vs. 7.1 ± 3.8, *p* = 0.03) scores, respectively (Table 2). Additionally, more patients among non-survivors had septic shock (6 vs. 0; *p* = 0.003) and COVID-19 infection (10 vs. 3; *p* = 0.001). All survivors had previously achieved microbiological response (10/10), while 4/10 deceased patients also had clearance of blood cultures. Between the two groups, no difference was noted regarding the use of Colistin, Aminoglycosides, Carbapenems, and Ampicillin–Sulbactam in therapeutic regimens. A significantly higher percentage in the survivor group received Fosfomycin (7 vs. 1; *p* = 0.02).

**Figure 1:**
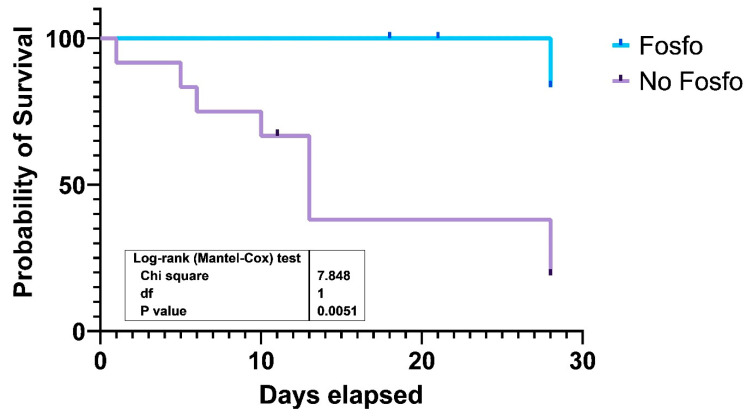
Kaplan–Meier curve for 28-day survival of patients treated with a fosfomycin-containing regimen or other antibiotic regimens in ICU patients with PDR *Acinetobacter baumannii* bacteremia. ICU: intensive care unit, PDR: pan-drug resistant.

The Kaplan–Meier curve for 28-day survival of patients treated with a fosfomycin-containing regimen or other antibiotic regimens is shown in Figure 1. Inclusion of fosfomycin in the combination therapeutic regimen was associated with significantly better survival as compared to non-fosfomycin-containing regimens (*p* = 0.0051).

## 4. Discussion

In the present series of ICU patients with PDR-AB primary bacteremia, high 28 d mortality of 50% was observed. Previous studies have demonstrated that crude mortality rates in patients with AB bacteremia vary between 30 and 76%, while in a recent multicenter study from Italy the ICU mortality rate in patients with carbapenem-resistant AB infections was 64.7% [12,13]. In our study, non-survivors presented higher illness severity on bacteremia diagnosis, higher rates of septic shock and COVID-19, which agrees with previous reports. Specifically, it has been previously shown that important risk factors for mortality in XDR-AB bacteremia are septic shock, associated with mortality rates over 90%, and COVID-19 with observed mortality over 75% [13,14].

With the emergence of colistin resistance in AB, our therapeutic armamentarium to treat these infections become very limited. Tigecycline is not an effective treatment option for bacteremias due to achievement of low serum concentrations of this drug associated with high mortality rates [10]. Novel beta-lactam beta-lactamase inhibitor combinations ceftazidime/avibactam, imipenem/cilastatin/relebactam and meropenem/vaborbactam are not active against AB strains. Amongst novel therapeutic options, eravacycline has demonstrated in vitro activity against AB, but regrettably the molecule did not reach predefined non-inferiority in clinical trials [15]. Cefiderocol, a novel siderophore cephalosporin, is currently the only β-lactam with displayed activity against AB, but this drug is not yet commercially available in our country. Therefore, in severe XDR and PDR infections, it is reasonable to use combination regimens to take advantage of antibiotic synergism. Different combinations of antibiotics have demonstrated a synergistic action against XDR-AB, such as colistin combined with rifampicin or tigecycline or sulbactam or vancomycin and carbapenems combined with colistin or sulbactam or aminoglycosides or rifampicin [16,17]. Previous studies have shown that the tigecycline–colistin combination was more synergistic than the tigecycline–rifampicin and colistin–rifampicin combination [18]. However, interpreting synergy data based on XDR-AB and extrapolating such data on PDR-AB has pitfalls. Especially considering traditional synergy definitions, synergy in vitro may only be present at clinically irrelevant concentrations [17,19]. In the present study, diverse combinations of antibiotics were used as salvage therapy. These combinations were mostly colistin-based in 17/20 patients (85%), since colistin constitutes the preferred backbone for such infections for many years. Tigecycline was used in 50%, fosfomycin in 40%, carbapenem and aminoglycoside in 35% and sulbactam (as high dose ampicillin/sulbactam 27 g/d) in 30% of patients. According to our results, the inclusion of fosfomycin in the therapeutic regimen was significantly associated with 28 d survival (*p* = 0.02).

Fosfomycin is an “old” antibiotic, discovered in 1969 as a phosphonic acid derivative. It is a water-soluble molecule with a low molecular weight (138.1 g/mol), with unique and unusually simple structure, containing an epoxy group that is essential for its antibacterial efficacy [20]. Fosfomycin is a bactericidal antibiotic that interferes with the first cytoplasmic step of bacterial cell wall biosynthesis, the formation of the peptidoglycan precursor UDP N-acetylmuramic acid [20]. Its unique mechanism of action makes cross-resistance uncommon and allows for synergy with other antibiotics. Numerous MDR gram-negative organisms are susceptible to fosfomycin and this, coupled with its excellent capacity for diffusion to various tissues, has resulted in several in vitro assessments of its antimicrobial capacity when combined with other agents [21]. Despite Acinetobacter’s genetic resistance to fosfomycin, mediated by efflux pumps, fosfomycin exerts a clinically useful synergy with diverse antibiotics against MDR-AB infections. The combination of fosfomycin with sulbactam has shown promising synergistic effects against carbapenem-resistant AB in vitro; synergy was observed in 74% isolates and no case of antagonism was reported [22]. Clinical data on the efficacy of intravenous fosfomycin in combination with other antibiotics against MDR-gram-negative infections are mainly derived from observational studies on enterobacterales and *P. aeruginosa* infections [23,24]. Regarding its clinical efficacy, as part of a combination regimen, against XDR-AB we were able to find only two studies. In the first study, ninety-four patients infected with carbapenem-resistant AB were randomized to receive colistin alone or colistin plus fosfomycin for 7 to 14 days; combination therapy with fosfomycin had a significantly more favorable microbiological response and a trend toward more favorable clinical outcomes and lower mortality [25]. The second recent prospective, observational, multicenter study, with 180 patients suffering from hospital-acquired pneumonia and ventilator-associated pneumonia from MDR-AB showed that the inclusion of fosfomycin in the treatment regimen led to a significant survival advantage [26].

A major limitation of our findings is that this is only a small case series study, retrospectively looking at the results of our experience and clinical practice with such difficult to treat infections. Patient characteristics and treatment regimens were very heterogeneous and the observed statistically significant results (e.g., regarding fosfomycin action) might have been affected by other diverse characteristics between survivors and non-survivors. With our study’s small sample size complex inferential statistics such as multivariate analysis with logistic regression is unreliable. Therefore, the present results need to be further checked in larger case series or prospective studies. Moreover, the potential synergistic effect of the antibiotics used in salvage combination regimens was not tested in vitro and was based on theoretical knowledge. On the other hand, the present case series enrolled only patients with PDR-AB primary bacteremia in a critical condition. Most studies with XDR or PDR-AB infections enroll patients with either hospital-acquired or ventilator-associated pneumonias, which represent the most common nosocomial AB infections [12,26]. However, isolation of AB from respiratory tract sample is not sufficient to establish its causal relationship with the infection and distinction between infection and colonization remains challenging. From this point of view, the present study presents data on true PDR-AB severe infections excluding the colonization confounder. In addition, as it is unlikely that a randomized controlled study will be performed on this topic, our results on the positive impact of fosfomycin-containing regimens may be of clinical value.

## 5. Conclusions

In conclusion, bacteremia caused by PDR-AB strains represents a challenge for physicians, considering the high rates of septic shock and mortality associated with this infection. In view of the increasing prevalence of PDR-AB infections in ICUs, its associated high rates of mortality and the lack of effective treatment options, we feel that there is an emerging need for our results on the positive impact of fosfomycin to be further validated in larger prospective studies.

## Figures and Tables

**Table 1:** Patients’ characteristics, treatment regimens and outcome.

Patient	AGE	SEX	CCI	COVID	APACHE II	Antibiotics Used	Microbiological Success	28-day Mortality
1	65	F	3	Yes	15	CST, TGC	Yes	No
2	53	M	1	Yes	24	CST, TGC	Yes	No
3	64	M	2	No	22	CST, SXT, FOS	Yes	No
4	64	M	4	No	10	CST, FOS	Yes	No
5	28	F	0	No	7	CST, AMS, FOS, AMK	Yes	No
6	76	M	6	No	8	CST, MEM	Yes	No
7	72	M	9	No	13	CST, TGC, FOS	Yes	No
8	64	F	6	No	25	TGC, AMS, FOS AMK	Yes	No
9	71	F	7	No	19	CST, TGC, FOS	Yes	No
10	36	F	0	Yes	20	CST, MEM, FOS, SXT, GEN	Yes	No
11	53	M	1	Yes	11	CST, TGC, AMS, SXT, AMK	Yes	Yes
12	60	M	2	Yes	17	CST, MEM, TGC, AMS	No	Yes
13	71	F	4	Yes	38	CST, TGC, FOS, AMK, SXT	Yes	Yes
14	59	M	1	Yes	40	CST, AMK	No	Yes
15	78	M	3	Yes	39	CST, PTZ	No	Yes
16	70	F	5	Yes	26	MEM, GEN	No	Yes
17	55	F	4	Yes	20	CST, MEM	No	Yes
18	62	F	2	Yes	28	MEM, AMS, TGC	No	Yes
19	55	F	1	Yes	25	CST, TGC, AMS	Yes	Yes
20	93	F	8	Yes	12	CST, MEM	Yes	Yes

M: male, F: female, CCI: Charlson comorbidity index, CST: Colistin, TGC: Tigecycline, SXT: Trimethoprim–Sulfamethoxazole, FOS: Fosfomycin, AMS: Ampicillin–Sulbactam, AMK: Amikacin, MEM: Meropenem, GEN: Gentamycin, PTZ: Piperacillin-Tazobactam, N/A: not applicable.

**Table 2:** Differences amongst survivors and non-survivors in ICU patients with PDR *Acinetobacter baumannii* bacteremia.

	28-day Mortality		Statistics
Characteristics	Survived (n = 10)	Died (n = 10)	*p*
Age years [Median (IQR)]	64 (48–71)	61 (55–72)	ns
Female Gender (%)	5 (50)	6 (60)	ns
Charlson Comorbidity Index mean ± SD	3.8 ± 3.1	3.1 ± 2.2	ns
APACHE II mean ± SD	16.3 ± 6.6	25.6 ± 10.8	0.032
SOFA mean ± SD	7.1 ± 3.8	11.4 ± 4.3	0.03
Septic Shock (%)	0	6 (60)	0.003
COVID (%)	3 (30)	10 (100)	0.001
≥3 antibiotics combination regimen (%)	6 (60)	5 (50)	ns
Colistin containing regimen (%)	9 (90)	8 (80)	ns
Tigecycline containing regimen (%)	5 (50)	5 (50)	ns
Fosfomycin containing regiment (%)	7 (70)	1 (10)	0.02
Aminoglycoside containing regimen (%)	3 (30)	4 (40)	ns
Carbapenem containing regimen (%)	2 (20)	5 (50)	ns
Ampicillin-Sulbactam containing regimen (%)	2 (20)	4 (40)	ns

ICU: intensive care unit, PDR: pan-drug resistant, SOFA: Sequential Organ Failure Assessment.

## Data Availability

The data presented in this study are available on request by the corresponding author.
